# Natural Course of Activated Phosphoinositide 3-Kinase Delta Syndrome in Childhood and Adolescence

**DOI:** 10.3389/fped.2021.697706

**Published:** 2021-07-19

**Authors:** Marketa Bloomfield, Adam Klocperk, Radana Zachova, Tomas Milota, Veronika Kanderova, Anna Sediva

**Affiliations:** ^1^Department of Immunology, 2nd Faculty of Medicine, Charles University Hospital in Motol, Prague, Czechia; ^2^Department of Pediatrics, 1st Faculty of Medicine, Charles University in Prague and Thomayer University Hospital, Prague, Czechia; ^3^Childhood Leukaemia Investigation Prague, Department of Paediatric Haematology and Oncology, 2nd Faculty of Medicine, Charles University and University Hospital in Motol, Prague, Czechia

**Keywords:** APDS, immunoglobulins, lymphoproliferation, immunodeficiency, infection, PI3K, activated phosphoinositide 3-kinase delta syndrome

## Abstract

Activated phosphoinositide 3-kinase delta syndrome (APDS), caused by mutations in PI3Kδ catalytic p110δ (*PIK3CD*) or regulatory p85α (*PIK3R1*) subunits, is a primary immunodeficiency affecting both humoral and cellular immunity, which shares some phenotypic similarities with hyper-IgM syndromes and common variable immunodeficiency (CVID). Since its first description in 2013, over 200 patients have been reported worldwide. Unsurprisingly, many of the newly diagnosed patients were recruited later in life from previously long-standing unclassified immunodeficiencies and the early course of the disease is, therefore, often less well-described. In this study, we report clinical and laboratory features of eight patients followed for APDS, with particular focus on early warning signs, longitudinal development of their symptoms, individual variations, and response to therapy. The main clinical features shared by our patients included recurrent bacterial and viral respiratory tract infections, gastrointestinal disease, non-malignant lymphoproliferation, autoimmune thyroiditis, and susceptibility to EBV. All patients tolerated vaccination with both attenuated live and subunit vaccines with no adverse effects, although some failed to mount adequate antibody response. Laboratory findings were characterized by dysgammaglobulinaemia, elevated serum IgM, block in B-cell maturation with high transitional B cells, and low naïve T cells with CD8 T-cell activation. All patients benefited from immunoglobulin replacement therapy, whereas immunosuppression with mTOR pathway inhibitors was only partially successful. Therapy with specific PI3K inhibitor leniolisib was beneficial in all patients in the clinical trial. These vignettes, summary data, and particular tell-tale signs should serve to facilitate early recognition, referral, and initiation of outcome-improving therapy.

## Introduction

Phosphatidyl-inositol-3-kinases (PI3Ks) belong to a family of enzymes encoded by the *PIK3* genes, ubiquitous in many different tissues throughout the human body, which regulate a wide spectrum of biological functions. A specific subgroup of this family, class IA PI3Ks, are master regulators of signaling within immune cells (1). Mutations in the *PIK3* genes were shown to cause an autosomal dominant immunodeficiency in humans (2). This so-called activated PI3K-delta syndrome (APDS) is caused by mutations in the *PIK3CD* (APDS1) and *PIK3R1* genes (APDS2) coding the catalytic p110δ subunit and the regulatory p85α subunit of the enzyme, respectively (3–5). These mutations exert an activating effect on the downstream signaling, particularly *via* increased phosphorylation of Akt and mTOR proteins, which in turn has a profound impact on cellular proliferation and differentiation (3–5).

Both lymphocyte lineages, as well as the cells of the innate immunity such as monocytes and dendritic cells, are affected in APDS (6). The germinal center reaction of B-lymphocytes, as well as their maturation, are disturbed in APDS patients, with reported increase in transitional B-lymphocytes and universal decrease of switched memory B-lymphocytes (3). Humoral immunity suffers in APDS patients, presumably due to impaired B-lymphocyte maturation and class switching, with high levels of IgM and low levels of IgG (IgG2 in particular) and IgA having been reported (2–4). The production of specific antibodies is also aberrant and may result in impaired vaccination response. T-lymphocytes are prone to premature senescence and cell death (2), with increased numbers of terminally differentiated cytotoxic CD8^+^CCR7^−^ T-lymphocytes characteristically overexpressing the senescence-related marker CD57 (4) and inhibitory receptors CD160, CD244, and PD-1 (7).

The characterization of APDS clinical and immunological phenotype was spearheaded by the seminal works of Elkaim et al. and Coulter et al. (8, 9). The phenotype of adult APDS patients is now known to be broadly heterogeneous, varying from asymptomatic state to combined immune deficiency. The hallmark features include recurrent or chronic sinopulmonary bacterial and viral infections, resulting in chronic lung damage, and generally benign lymphoproliferative disease. Particular susceptibility to herpetic viruses has been recorded, as well as increased incidence of autoimmune/autoinflammatory complications, and hematopoietic malignancies. On the other hand, the phenotype of pediatric patients and clinical course preceding the diagnosis is less explored. Because of the beneficial effect of early treatment with immunoglobulin replacement therapy (IgRT), antibiotic, anti-virotic, and antifungal prophylaxis, as well as the availability of targeted therapeutics, such as broader-spectrum mechanistic target of rapamycin (mTOR) inhibitors and specific PI3K inhibitors, the timely diagnosis of the disease is of utmost importance.

In this study, we present the summary of clinical and laboratory findings as well as short case vignettes of eight patients with APDS from the Czech Republic, four of whom are children, with particular focus on the early course of the disease. This material should serve to facilitate the early referral of candidate patients to a specialist for targeted genetic evaluation, treatment, and outcome improvement.

## Patients and Methods

### Patient Cohort

All patients are followed at the Department of Immunology, Second Faculty of Medicine, Charles University and University Hospital in Motol, Prague, Czech Republic, at the time of manuscript preparation. Written informed consents with collecting and publishing of clinical and laboratory data were provided by the participants or their legal guardians/next of kin in accordance with the Declaration of Helsinki and local ethical requirements. Data on demographics, clinical manifestations, laboratory features and other investigations, therapeutic management, and outcomes were collected retrospectively from medical records of the patient or obtained *via* patient/parent interview.

### Investigations

Genetic testing was performed using either Sanger sequencing or whole-exome sequencing verified by Sanger sequencing, as described previously (10).

Immunological laboratory data were obtained using routine in-house methods. For immunocyte phenotyping, published reference values (11, 12) and in-house controls were used. Commercially available ELISA assays were used to measure the SARS-CoV-2 IgA and IgG spike protein antibodies (EUROIMMUN, Lübeck, Germany). The chest Xray images, computed tomographic scans, and histopathology specimens were evaluated by specialized radiologists and pathologists, respectively.

## Results

Eight patients with genetically confirmed APDS, three males and five females, from five non-consanguineous Czech families of Caucasian ethnicity, were included in this study (Table 1). The current median age is 15 years (range 6–37 years). Seven patients (P1–P7) harbor a previously described missense mutation p.E1021K (c.3061G>A) in *PIK3CD*, and one patient (P8) carries the splice site mutation c.1425+1G>C in the *PIK3R1*. Six out of eight patients have one or more suspected or confirmed relative(s) with APDS.

**Table 1:** Clinical data, part 1.

	**P1**	**P2**	**P3**	**P4**	**P5**	**P6**	**P7**	**P8**	**Overall**
Mutation gene	*PIK3CD*	*PIK3CD*	*PIK3CD*	*PIK3CD*	*PIK3CD*	*PIK3CD*	*PIK3CD*	*PIK3R1*	
Mutation position	p.E1021K	p.E1021K	p.E1021K	p.E1021K	p.E1021K	p.E1021K	p.E1021K	c.1425+1G>C	
Mutation comment	Missense, exon 23	Missense, exon 23	Missense, exon 23	Missense, exon 23	Missense, exon 23	Missense, exon 23	Missense, exon 23	Splice site, resulting in exon 11 deletion	
Sex	M	F	M	F	F	M	F	F	
Suspect family history	Sister Sister's son	Brother Son	Mother Uncle	Father	None	Mother	Son	-	6/8 (75%)
Age at evaluation	30 years	37 years	8 years	17 years	25 years	6 years	31 years	13 years	Median 15 years (range 6–37 years)
Age at onset of infections	1 month	2 years	4 years	2 years	12 months	8 months	12 months	12 months	Median 12 months (range 1 month−4 years)
Age at onset of lymphoproliferation	1 year	3 years	5 years	2 years	2 years	8 months	5 years	5 years	Median 2.5 years (range 8 months−5 years)
Age at diagnosis Year of diagnosis	23 years 2013	30 years 2013	2 years 2013	11 years 2014	21 years 2016	5 years 2019	30 years 2019	7 years 2014	Median 16 years (range 2–30 years)
Diagnostic delay	22 years	28 years	0 year	7 years	20 years	4 years	29 years	6 years	Median 13.5 years (range 0–29 years)

*F, female; M, male*.

### Clinical Manifestations

**Figure 1:**
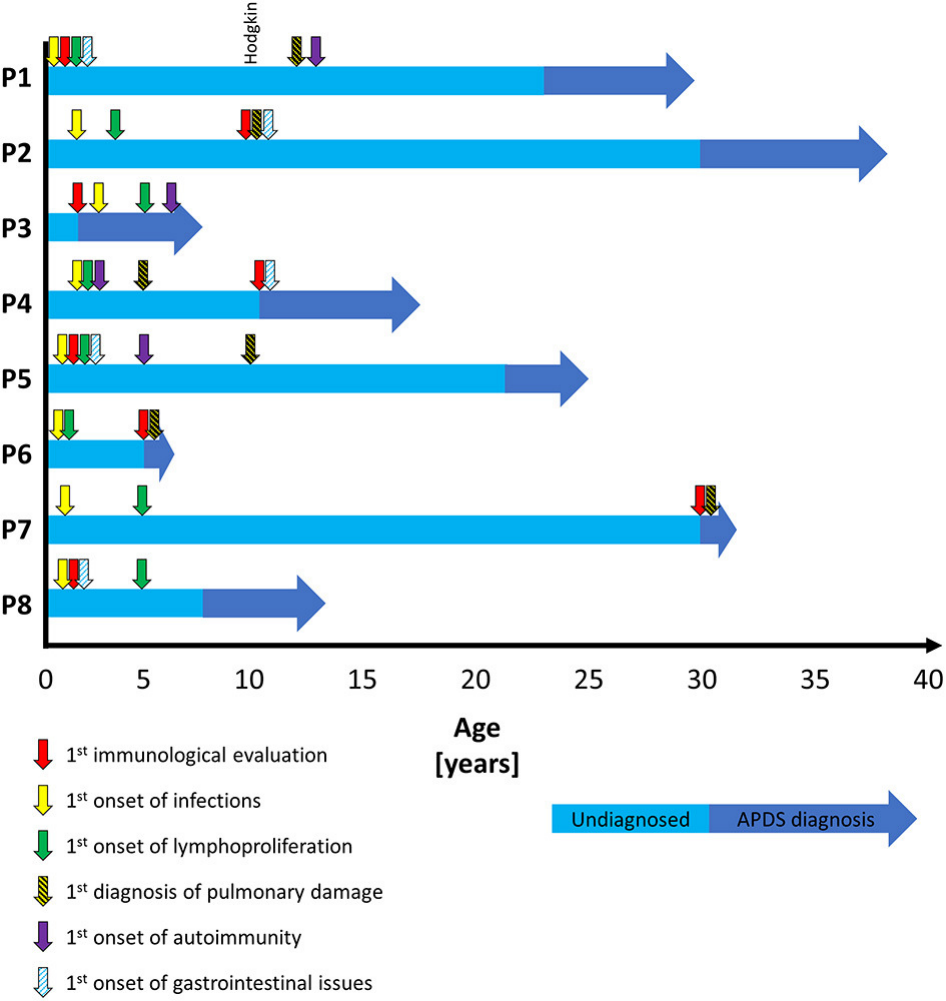
Patient vignette timeline.

All patients had become symptomatic prior to their fourth year of age (median 12 months, range 1 month-4 years), seven out of eight patients manifested within the first 2 years of life. The median age at APDS diagnosis was 16 years (range 2–30 years). Four patients were diagnosed in adulthood. The median diagnostic delay was 13.5 years (range 0–29 years), with the main predictor being the age at first publication describing the disease (Table 1 and Figure 1).

The dominant symptoms were frequent upper (rhinitis, sinusitis, otitis, or tonsillitis) or lower (bronchitis and pneumonia) respiratory tract infections and unexplained chronic lymphoproliferation in all patients (Table 2). The detected pathogens were mostly common bacterial strains, e.g., *Streptococcus, Staphylococcus, Haemophilus, Neisseria* species, and common respiratory viruses (unspecified).

**Table 2:** Clinical data, part 2.

	**P1**	**P2**	**P3**	**P4**	**P5**	**P6**	**P7**	**P8**	**Overall**
Respiratory tract infections	Upper and lower	Upper and lower	Upper and lower	Upper and lower	Upper and lower	Upper and lower	Upper and lower	Upper and lower	8/8 (100%)
RTI pathogens	*HI, Strepto, Pneumo, Staphylo*, and viruses	*HI, Strepto, Pneumo, Staphylo, Bran.cat., Morg.morg., Candida alb.*, and viruses	*HI, Strepto, Pneumo, Staphylo, Neisseria, Morg.morg., H1N1 inf.*,and viruses	*HI, Strepto, Pneumo, Adenovirus*, and viruses	*HI, Strepto, Pneumo, Staphylo, Neisseria, Candida alb.*, and viruses	*Strepto, Staphylo, Pseud. oryzi.*, and viruses	*HI, Strepto, Pneumo, Staphylo*, and viruses	*Strepto, Staphylo, Neisseria*, and viruses	Bacterial 8/8 (100%). viral 8/8 (100%), and fungal 2/8 (25%)
Other infections	*Coxsackie B* pericarditis, Lyme disease, *H.pylori* gastritis, and COVID-19	EBV, sporadic, and mammary abscess	*Staph. aureus* dermatitis	episodic EBV, EPEC, and enterobiosis	episodic EBV, purulent conjunctivitis, and COVID-19	EBV, sporadic, and COVID-19	EBV, sporadic, recurrent labial HSV, and COVID-19	Recurrent labial HSV and purulent conjunctivitis	EBV 5/8 (62.5%), HSV 2/8 (25%), and COVID-19 4/8 (50%)
Lymphoproliferation	LAD, HS-megaly, RT LH, and GIT LH	LAD, tonsils, RT LH, and GIT LH	LAD, adenoids, and tonsils	LAD, adenoids, tonsils, HS-megaly, RT LH, and GIT LH	LAD, adenoids, tonsils, S-megaly, and RT LH	LAD, adenoids. tonsils, HS-megaly, RT LH, and GIT LH	LAD, adenoids, S-megaly, and T-cell infiltration of skin	LAD, adenoids, and tonsils	8/8 (100%)
Histopathology	**Cervical LN**, 90% lymphocytes, predominantly CD4 T-cells, well-formed GCs, no malignant changes, EBV–	**Cervical LN**, polyclonal lymphoproliferation, no malignant changes	**Cervical LN**, disrupted architecture with well-formed GCs, MZ hyperplasia, non-clonal, predominantly B cell expansion with kappa light chains restriction, EBV–	**Bronchial wall biopsy** non-malignant lymphocytic infiltrate, abundant lymphatic follicles, EBV–	**Cervical LN** atypical lymphoproliferation, predominantly CD4+, no malignant changes, EBV+	**Tonsils** atypical lymphoproliferation, non-malignant, EBV–	**Cutaneous infiltration** atypical lymphoproliferation, predominantly CD3+ infiltrate, non-malignant, EBV–	**Cervical LN** follicular hyperplasia with paracortical expansion, well-formed GCs, no malignant changes	Non-malignant lymphocytic proliferation 8/8 (100%)

*BPN, bronchopneumonia; Bran.cat., Branhamella cattarhalis; CS, corticosteroids; EPEC, enteropathogenic E. Coli; episodic, detected on multiple occasions; GC, germinal center; GIT, gastrointestinal tract; HI, Haemophilus influenzae; H1N1 inf., H1N1 influenza; HS-megaly, hepatosplenomegaly; LAD, lymphadenopathy; LH, lymphoid hyperplasia; LN, lymph node; Morg.morg., Morganella morgani; MZ, marginal zone; NA, not available; Pneumo, Streptococcus pneumoniae; Pseud.oryzi., Pseudomonas oryzihabitans; RT, respiratory tract; RTI, respiratory tract infection (upper RTI=rhinitis, sinusitis, otitis, and tonsilitis; lower RTI=bronchitis and pneumonia); S-megaly, splenomegaly; Staphylo, Staphylococcus spp., Strepto, Streptococcus spp., sporadic, detected only once*.

Two patients (P7, P8) experienced recurrent HSV infection. EBV infection was clinically uneventful in 3/8 patients (38%; P2, P6, and P7), and 2/8 patients (25%; P4 and P5) were found to have a low-level EBV viremia or tissue polymerase chain reactions (PCR) EBV positivity on multiple separate occasions. Three out of eight patients (38%; P5, P6, and P7) had persistent positivity of IgM to viral-capsid antigen (VCA) while failing to produce Epstein–Barr nuclear antigen 1 (EBNA) antibodies suggesting a defect in EBV clearance. *Candida albicans* was detected on a singular occasion during RTI in two patients (25%; P2 and P5); otherwise, no particular susceptibility to fungal pathogens was observed. Four out of eight patients (50%; P1, P5, P6, and P7) contracted SARS-CoV-2 in 2021, yet suffered only mild symptoms. All but one (P1) mounted an adequate virus-specific IgA and IgG response. Six patients (75%) received live attenuated BCG vaccine, and all patients received live measles, mumps, and rubella vaccines (MMR) without any adverse reactions.

The persistent or recurrent enlargement of secondary lymphoid organs had developed in all patients before the age of 5 years (median 2.5 years, range 8 months to 5 years). All patients manifested localized or diffuse lymphadenopathy; 7/8 patients (88%) had enlarged tonsils or adenoids; 4/8 patients (50%) had expanded mucosa-associated lymphatic tissues in the gut; and 5/8 (63%) in the bronchi; 5/8 (63%) had splenomegaly. All patients had at least one tissue biopsy, which revealed abundant polyclonal lymphocytic expansion in all samples (lymph nodes, tonsils, bronchial wall, and skin) described by the histopathopathologist as atypical but benign in most cases; EBV was detected in one specimen only (lymph node in P5).

One patient (P1) developed Hodgkin lymphoma (EBV negative) at the age of 11 years and achieved full remission with standard treatment. No other malignancies were observed within the cohort. One patient (P7) developed a progressive infiltrative skin lesion of the neck at 24 years resembling a cutaneous lymphoma, which was, however, evaluated as non-malignant.

Gastrointestinal involvement, presenting as abdominal discomfort, was reported by 5/8 patients (63%; P1, P2, P4, P6, and P8) (Table 3). Additionally, 3/8 patients (38%; P1, P6, and P8) experienced chronic diarrhea onsetting prior to the second year of life; however, only one patient (P1) failed to thrive. The endoscopy findings uniformly demonstrated intestinal lymphonodular hyperplasia, histologically specified as non-malignant, predominantly CD3+ lymphocytic expansion in lamina propria.

**Table 3:** Clinical data, part 3.

	**P1**	**P2**	**P3**	**P4**	**P5**	**P6**	**P7**	**P8**	**Overall**
Pulmonary damage	Bronchiectasis at 13 years, fibrosis, and COPD	Bronchiectasis at 10 years, fibrosis, and ACOS	-	Bronchiectasis at 5 years, fibrosis, and lobe resection	Bronchiectasis at 10 years, atelectasis, and mixed ventilatory impairment	Bronchiectasis at 5 years, atelectasis, and mixed ventilatory impairment	Bronchiectasis at 30 years	-	6/8 (75%)
GIT involvement	Abdominal discomfort, diarrhea, malabsorption	Abdominal discomfort	-	Abdominal discomfort	-	Abdominal discomfort, diarrhea	-	Abdominal discomfort and diarrhea	5/8 (62.5%)
Other	-	Allergic rhinitis, dermatitis	Conductive hearing loss, delayed speech development	Systemic hypertension	Conductive hearing loss	-	-	Autism and spectrum disorder	-
Malignancy	Hodgkin lymphoma, EBV-	-	-	-	-	-	-	-	1/8 (12.5%)
Autoimmunity	Thyroiditis	-	Thyroiditis	Thyroiditis	Thyroiditis	-	-	-	4/8 (50%)
Treatment (age at initiation)	IVIg and SCIg (15 months), ATB prophylaxis (7 years), Rapamycin (25 years), Leniolisib study (26 years)	SCIg (19 years), ATB prophylaxis (19 years)	SCIg (7 years)	CS (3 years), SCIg (11 years), ATB prophylaxis (11 years), Leniolisib study (16 years)	SCIg (8 years), ATB prophylaxis (8 years), antifungal prophylaxis (8 years), Rapamycin (21 years), Leniolisib study (23 years)	CS (12 months), SCIg (5 years), ATB prophylaxis (5 years)	SCIg (30 years), Leniolisib study (31 years)	SCIg (2.5 years), ATB prophylaxis (2.5 years), antifungal prophylaxis (2.5 years), and antiviral prophylaxis (6 years).	SCIg 8/8 (100%), ATB prophylaxis 6/8 (75%), antiviral prophylaxis 1/8 (12.5%), antifungal prophylaxis 2/8 (25%), CS 1/8 (12.5%), Rapamycin 2/8 (25%), Leniolisib 4/8 (50%)
Vaccination	inactivated, MMR, BCG	inactivated, MMR, BCG	inactivated, MMR	inactivated, MMR, BCG	Inactivated, MMR, and BCG	Inactivated, MMR	Inactivated, MMR, BCG, and influenza	Inactivated, MMR, and BCG	-

*ACOS, asthma-COPD overlap syndrome; ATB, antibiotics; BCG, bacillus Calmette–Guérin; CS, corticosteroids; COPD, chronic obstructive pulmonary disease; GIT, gastrointestinal tract; IVIg, intravenous immunoglobulin, MMR, measles, mumps, rubella; SCIg, subcutaneous immunoglobulin*.

Lung damage was noted in 6/8 patients (75%; P1, P2, and P4–P7), median age at detection was 10 years (range 5–30 years). The common findings were bronchiectasis (75%; P1, P2, and P4–P7). Prior to genetic diagnosis, ventilatory impairment was found in four patients (50% from the cohort); chronic obstructive pulmonary disease (COPD) in P1, asthma/COPD overlap syndrome in P2, and mixed ventilatory impairment in P5 and P6. Pulmonary fibrosis was detected in 3/8 patients (38%; P1, P2, and P4), and in one patient (P4) lobectomy had to be performed.

Four patients developed autoimmune thyroiditis (50%; P1, P3, P4, and P5) at a median age of 6 years (range 2–13 years). Two patients (P1 and P4) had transiently positive pANCA autoantibodies; no other autoantibodies or clinical autoimmune phenomena were present in any of the patients.

Among other clinical manifestations, pericarditis caused by *Coxsackie* infection, Lyme disease-associated Bell's palsy, mammary abscess, early staphylococcal dermatitis, allergic rhinitis, and systemic hypertension were found in one patient each (P1, P1, P2, P3, P2, and P4, respectively). Conductive hearing impairment was diagnosed in two patients (P3 and P5) and resolved with adenotomy. As for neurodevelopmental morbidity, one patient experienced delayed speech development (P3) and one patient (P8) was diagnosed with autism spectrum disorder.

### Immunology Investigations

**Figure 2:**
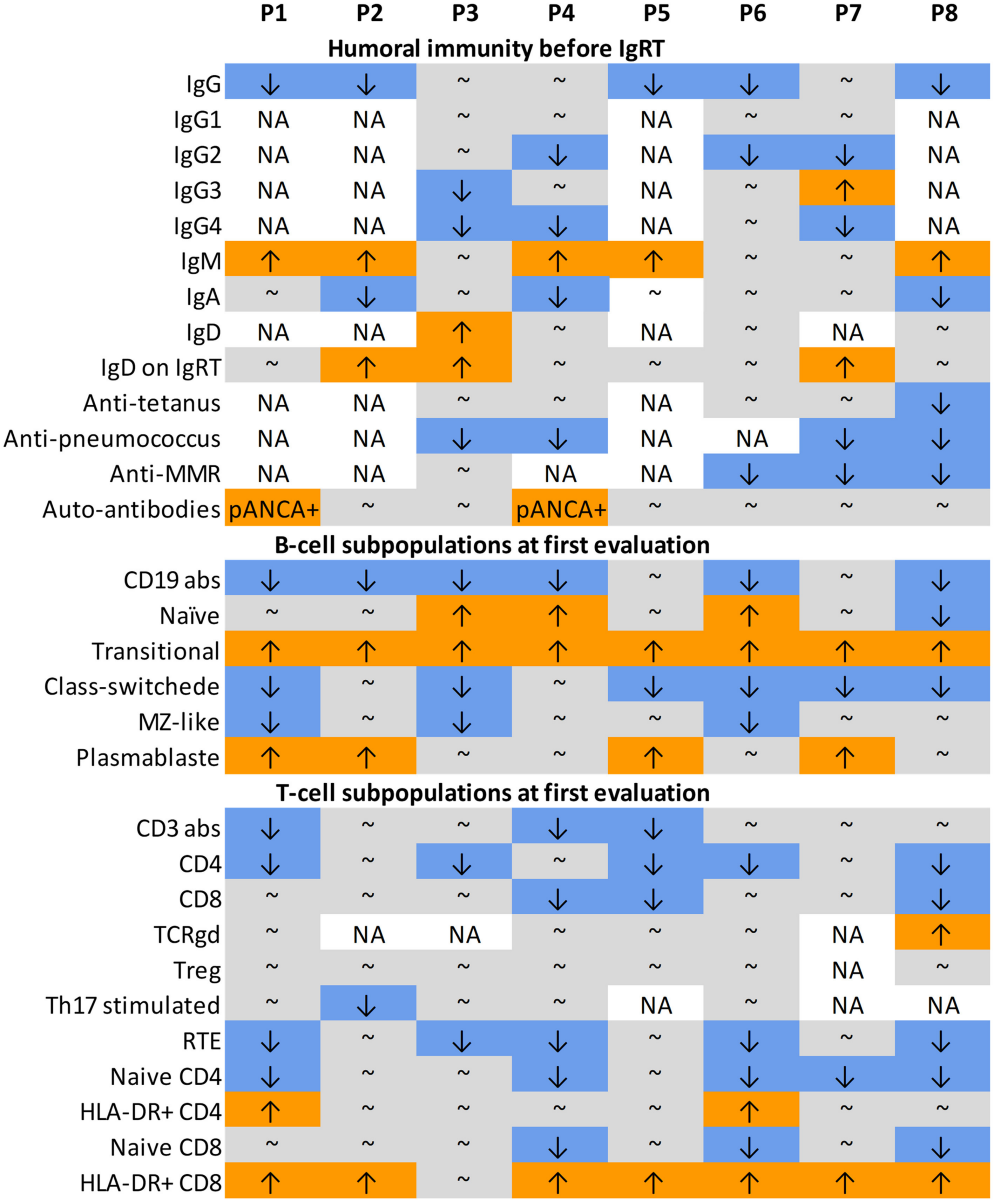
Laboratory data. ↑ value above normal age-matched range; ↓ value below normal age-matched range; ∽ value within normal age-matched range; abs, absolute counts; IgRT, immunoglobulin replacement therapy; MMR, mumps, measles, morbilli; MZ-like, marginal zone-like; NA, not available; pANCA, antineutrophil cytoplasmic antibody, perinuclear pattern; RTE, recent thymic emigrants.

The initial immunological evaluation, performed in all patients due to recurrent infections, revealed IgG hypogammaglobulinemia in 5/8 patients (63%), which had been present since childhood and led to an early immunoglobulin substitution therapy (Figure 2). Elevation of IgM was found in 5/8 patients (63%). Concurrently, IgA was low in 3/8 patients (38%). Most IgG subclasses were also low, reflective of the decreased total IgG, but we saw an isolated decrease in IgG2 with normal IgG in two patients, suggesting that such findings should prompt more detailed immunologic work-up. Interestingly, 3/8 patients (38%) had elevated IgD levels, which normalized in one patient on leniolisib (P7). All patients failed to respond adequately to pneumococcal vaccines, either protein based or polysaccharide. Response to live vaccines (MMR) was impaired in all but one (P3). Four patients tested prior to IgRT responded well to tetanus vaccine. B-cell immunophenotype was consistent between all patients. Low overall B-cell numbers (6/8 patients, 75%), high naïve (3/8, 38%), and transitional (8/8, 100%) but low class switched (6/8, 75%) and marginal zone-like (3/8, 38%) B cells illustratively document a block in B-cell maturation. Interestingly, 4/8 (50%) patients also had high circulating plasmablasts.

Similarly, the T-cell phenotype was also highly consistent across the cohort. While the overall T-cell counts were only decreased in 3/8 (37.5%) patients, a decrease in CD4 T-cells was more typical [seen in 5/8 (63%) of patients], and T-cell differentiation pattern was markedly skewed toward activated CD8 T-cells 7/8 (87.5%). On the other hand, the naïve CD4, naïve CD8, and recent thymic emigrants were decreased in 5/8 (63%), 3/8 (38%), and 5/8 (63%), respectively. No major changes were seen in the gamma delta, regulatory, and Th17 T-cell populations.

### Management Strategies

All patients received a myriad of systemic antibiotics for RTI during their early childhood. Six out of eight patients (75%) were eventually started on prophylactic antibiotic regimen (trimethoprim/sulfamethoxazole; median age at initiation 7.5 years, range 2.5–19 years); median time from first symptoms to initiation was 7 years (range 2–17 years); two patients (25%) received prophylactic antifungal compound (itraconazole; P5 for recurrent *Candida sp*. RTI, achieving remission of fungal infections, and P8 without prior fungal susceptibility); one patient (P8) received prophylactic acyclovir for recurrent labial HSV, which prevented further HSV reactivations. Regardless of the serum immunoglobulin levels, all patients received IgRT (median age at initiation 7.5 years, range 15 months to 30 years; median time from first symptoms 7 years, range 1–29 years). Five out of six patients started IgRT at the same time as the antimicrobial prophylaxis; therefore, the effect of each strategy may not be assessed individually in our cohort. However, all five patients achieved mitigation of the frequency of infections, and lymphoproliferation regressed in four out of six patients (all except P2 and P8). Moreover, the three patients who were at least for some time solely on IgRT (P1-15 months to 7 years, and P3 and P7-no antibiotic), all experienced at least a partial reduction in infectious susceptibility while on IgRT, whereas the alleviation of the lymphoproliferation was only achieved in one. Systemic oral corticosteroids were used in P4 (to control the lymphadenopathy and hepatosplenomegaly, with only partial and short-term effect) and P6 (due to intestinal lymphonodular hyperplasia with minimal effect).

mTOR inhibitor rapamycin (sirolimus) was used in two patients (25%; P1 and P5), achieving some, but not ample improvement of infectious susceptibility and sequelae of lymphoproliferation. In P1, no changes in immunoglobulin levels were noted during the 5-month treatment, while the transitional B cells decreased, and the naïve CD4 increased. In P5, rapamycin evoked no immunophenotype change during the 7 months of treatment.

Four patients (50%; P1, P4, P5, and P6) were enrolled into a clinical study evaluating the safety and efficacy of the specific PI3K inhibitor leniolisib, which is ongoing at the time of drafting this manuscript (more information at https://clinicaltrials.gov/ct2/show/NCT02435173). In general, the treatment benefited all the patients, particularly in reducing the lymphoproliferation.

The autoimmune thyroiditis was succesfully managed by hormone replacement therapy alone in all patients.

Two patients (P2 and P7) achieved spontaneous conceptions, uneventful pregnancies, and delivered termed eutrophic infants (P2 while on IgRT and P7 prior to IgRT).

The clinical and laboratory data of all patients are shown in Tables 1–3, and Figures 1, 2. Supplementary Table 1 shows detailed laboratory results. Clinical vignettes of the patients are presented below.

### Patient Vignettes

#### Patient 1

P1 is a 30-year-old male with APDS1 (p.E1021K). He has been suffering from recurrent RTI since 1 month of age, developed persistent non-malignant lymphatic tissue hyperplasia (i.e., hepatosplenomegaly, multiple site lymphadenopathy, and gastrointestinal lymphoid hyperplasia), chronic diarrhea, and failure to thrive in the first year of life. He was treated for *Helicobacter pylori*-positive chronic gastritis and, at 6 years of age, underwent appendectomy. At 10 years, he suffered exsudative pericarditis caused by *Coxsackie B4* virus. At 11 years, nodular sclerosis classical Hodgkin lymphoma was diagnosed. Full remission was achieved with standard treatment. After severe pneumonia at 13 years of age, he was found to have reduced lung functions, bronchiectasis, and fibrosis on chest high-resolution computed tomography (HRCT). At the same time, hypothyroidism was diagnosed. At 15 years of age, he contracted Lyme disease and developed bilateral Bell's palsy. At 30 years, he suffered SARS-CoV-2 infection with only mild respiratory symptoms and self-limiting fever. He mounted only a weak IgA antibody response (no specific IgG), reverting to seronegativity within 2 months.

P1 was evaluated by an immunologist for the first time at 1 year of age. The available medical record states severe IgG, IgA hypogammaglobulinemia, and increased IgM. IgRT achieved only a partial reduction in infectious susceptibility. At 7 years of age, prophylaxis with co-trimoxazole was initiated, markedly reducing the RTI. No impact on lymphatic tissue enlargement was observed. Later in life, the cellular immunophenotype of P1 was disease characteristic, with the exception of extremely high circulating plasmablasts. Upon the diagnosis of APDS at 23 years of age, rapamycin was introduced, achieving only minor clinical improvement. Less than 1 year later, the patient was enrolled in a clinical study with PI3K inhibitor leniolisib.

P1 is of normal intellect, achieved basic education, and now works as a manual laborer.

The sister of the patient (P2, same parents) and her son (P3) both carry the identical mutation in *PIK3CD*. Curiously, the parents of P1 and P2 are healthy and do not carry the mutation in their peripheral blood cells.

#### Patient 2

P2 is a 32-year-old sister of P1 with APDS1 (p.E1021K). She has been experiencing frequent RTI since 2 years of age and persistent non-malignant lymphatic tissue hyperplasia since 3 years of age. After her first pneumonia at 10 years, she was found to have signs of asthma and chronic obstructive pulmonary disease overlap syndrome, and multiple bronchiectasis and small airways disease were seen on HRCT. Additionally, allergic rhinitis with IgE sensitization to grass pollen was diagnosed. At 28 years of age, she developed a mammary abscess. Her pregnancy at the age of 29 years and the delivery of a termed newborn (P3) were uneventful. At 30 years of age, GIT endoscopy was performed for complaints of abdominal discomfort, detecting prominent mucosal lymphoid hyperplasia.

P2 was evaluated by an immunologist for the first time at 10 years. Low serum IgG, IgA, and increased IgM were noted. More detailed work-up later in life revealed typical APDS cellular immunophenotype; however, the preserved T-cell counts, maturation, and normal class-switched memory B cells may reflect the patient's milder phenotype compared with P1.

As the preliminary diagnosis of hyper-IgM syndrome was established at 19 years of age, IgRT and antibiotic prophylaxis with cotrimoxazole were initiated, achieving a major reduction of RTI and deceleration of lung function deterioration. Similarly to P1, no impact on lymphadenopathy or GIT symptoms was observed. The diagnosis of APDS was established at 30 years of age along with her son and her brother.

The patient achieved basic education and worked shortly as maintenance staff. She currently receives a disability pension and is treated for alcohol addiction.

#### Patient 3

P3 is an 8-year-old son of P2 with APDS1 (p.E1021K). At 4 years of age, he developed chronic rhinitis and frequent otitis media (common pathogens and H1N1 influenza virus), requiring the insertion of ventilation tubes, and loci of dermatitis. The lymphoid hyperplasia manifested at 5 years of age (i.e., cranial, cervical, axillary lymphadenopathy, hypertrophic adenoids, and tonsils). Lymph node biopsy revealed well-formed germinal centers with non-clonal, predominantly B-cell expansion. At 7 years of age, the patient developed subclinical autoimmune thyroiditis.

P3 was evaluated by an immunologist for the first time at 2 years of age, when decreased IgG and IgA were detected. However, when re-assessed at 7 years, he presented with normal total IgG and IgA, decreased IgG3, IgG4 subclasses, and low response to conjugated pneumococcal antigen. Even though the hypogammaglobulinemia normalized by 7 years, the patient was started on subcutaneous IgRT, which achieved a marked amelioration of infectious susceptibility and almost complete regression of lymphadenopathy. His cellular immunophenotype was disease characteristic; the normal activation status of T cells may reflect the early stage of the disease.

The speech development of this patient was delayed; otherwise, his psychomotor development was undisturbed. He attends secondary school, achieving average results.

#### Patient 4

P4 is a 17-year-old female diagnosed with APDS1 (p.E1021K). Her father died at 31 years due to multi-organ failure after a history of RTI, pulmonary fibrosis, chronic hepatitis, nephrotic syndrome, and systemic amyloidosis. She manifested at 2 years of life with EBV and enteropathogenic *E. coli* co-infection accompanied by severe lymphadenopathy, hepatosplenomegaly, and subclinical hypothyroidism. She continued experiencing frequent RTI (common pathogens) and underwent several lymphadenectomies, adenoidectomy, and tonsillectomy by the fifth year of age, histologically assessed as benign lymphoproliferation. At 5 years, pulmonary fibrosis was noted on chest HRCT and EBV DNA was detected in bronchoalveolar lavage; at 9 years, a lobectomy was performed for severe bronchiectasis and atelectasis, due to contiguous non-neoplastic lymphocytic bronchial infiltrate. EBV-positive gastrointestinal lymphonodular hyperplasia was found at 11 years as part of investigation for abdominal discomfort just prior to the APDS diagnosis. At the same time, she was started on ACE inhibitors for systemic hypertension.

Prior to the first evaluation of P4 by an immunologist, she received several courses of steroids in an attempt to control the lymphoproliferation with only a short-term effect. At 11 years of age, she was found to have decreased IgG2, IgG4, and IgA levels, increased IgM, and diminished response to polysaccharide antigens. Cellular immunophenotype was disease characteristic. IgRT and prophylactic antibiotic diminished the infectious susceptibility and partially ameliorated the lymphoproliferative disease. At 16 years of age, she entered a clinical study with PI3K inhibitor leniolisib.

P4 is of normal intellect, currently attends grammar school, and achieves good results.

#### Patient 5

P5 is a 25-year-old female with APDS1 (p.E1021K). She has suffered from RTI since 12 months (common pathogens and *Candida albicans*) necessitating early insertion of ventilation tubes and adenoidectomy for hearing impairment. At 2.5 years of age, she was hospitalized for EBV infection with marked hepatosplenomegaly and diffuse lymphadenopathy. At 5 years, autoimmune thyroiditis was diagnosed. At 8 years, tonsillectomy and cervical lymphadenectomy were performed, showing atypical but polyclonal lymphoproliferation with EBV positivity. Later, chronic lobar atelectasis, underlaid by pronounced bronchial lymphonodular hyperplasia, and bronchiectasis were noted, manifesting as chronic cough and steroid-resistant mixed ventilatory impairment. At 25 years, she was found to have positive SARS-CoV-2 IgA and IgG antibodies, while unaware of any past symptoms of the disease.

P5 was evaluated by an immunologist for the first time at 10 years of age. Low IgG and increased IgM were noted. By 20 years of age, her immunophenotype was disease characteristic, and the diagnosis of APDS was established. She had a persistent anti-EBV viral capsid antigen (VCA) IgG and IgM positivity but absent Epstein–Barr nuclear antigen 1 (EBNA) IgG.

IgRT and prophylaxis with cotrimoxazole and itraconazole were started at 8 years of age, partially reducing the frequency of RTI and lymphoproliferation. Upon the diagnosis of APDS at 21 years, rapamycin was started, further ameliorating the infectious susceptibility and lymphoproliferation. At 23 years of age, she entered a clinical study with PI3K inhibitor leniolisib.

P5 is of normal intellect, achieved high school education, and currently works as a receptionist.

#### Patient 6

P6 is a 6-year-old son of P7 diagnosed with APDS1 (p.E1021K). He has suffered from recurrent RTI since 8 months of age (common bacterial and viral pathogens). At 12 months, EBV infection manifested with a prolonged febrile episode with mesenteric lymphadenopathy, which was followed by chronic diarrhea, and persistent benign lymphoproliferation (lymphadenopathy, hepatosplenomegaly, and intestinal lymphonodular hyperplasia). At 4 years, his adenoids and tonsils were removed, displaying profound non-malignant EBV-negative lymphocytic infiltration. Upon the detection of mixed ventilatory impairment at 5 years of age, he was found to have multiple bronchiectasis and loci of atelectasis on CT, likely due to the pronounced bronchial lymphonodular hyperplasia. At 6 years, he contracted SARS-CoV-2 virus, experienced only mild cough, and mounted a normal antibody response (single measurement).

P6 was evaluated by an immunologist for the first time at the age of 5 years. He was found to have mildly decreased IgG and abrogated response to MMR vaccine. His cellular immunophenotype was characteristic for APDS, which was established shortly afterward. Unlike P3, P6 displayed marked activation of CD4 and CD8 -cells. Despite a long-term positivity of VCA IgM and IgG, he failed to mount an EBNA response, yet EBV viremia was never detected in the peripheral blood.

Cotrimoxazole prophylaxis and IgRT were introduced at 5 years with an excellent effect on infectious susceptibility but only partially reducing the lymphoproliferation.

The patient has normal intellect and currently attends primary school.

#### Patient 7

P7 is a 31-year-old mother of P6 diagnosed with APDS1 (p.E1021K). She has been suffering from frequent RTI from early infancy (common pathogens) and experienced occasional labial HSV. A significant decrease in RTI frequency was noted in her third decade of life; however, she retrospectively admitted having experienced chronic cough. At 30 years, chest HRCT showed prominent bronchiectasis and mediastinal lymphadenopathy. The abnormal lymphoproliferation was apparent since early childhood, necessitating adenectomy and cervical lymphadenectomy at 7 and 12 years, respectively. Since 24 years of age, she has been followed by oncologists for suspected cutaneous lymphoma of the neck; however, repeated biopsies revealed non-neoplastic chronic lymphoproliferation. Concurrently, the whole-body positron emission tomography CT scan showed diffuse lymphadenopathy and mild splenomegaly. At 31 years, she contracted SARS-CoV-2 virus, experienced only mild respiratory symptoms and nausea, and mounted an adequate antibody response (single measurement). P7 had two uncomplicated pregnancies and delivered two children *via* cesarean section at 24 and 25 years of age (P6 and another healthy boy).

P7 was evaluated by an immunologist for the first time at 30 years of age, when her son was diagnosed with APDS. She was found to have low IgG2, IgG4, and low post-vaccination response to polysaccharide antigens and MMR. Her peripheral lymphocytes displayed a similar phenotype to her sons', although less pronounced, with a surprising elevation of plasmablasts. Also, similar to her son, she failed to mount an EBNA response to EBV, despite a long-term positivity of VCA IgM and IgG.

She was started on IgRT and, shortly afterward, entered into the clinical study with PI3K inhibitor leniolisib.

She received a bachelor's degree and works as an office worker.

#### Patient 8

P8 is a 13-year-old female with APDS2 (c.1425 + 1 G > C). In her early infancy, she presented with chronic rhinitis (common pathogens); in her second year of life she started experiencing more severe RTI, along with purulent conjunctivitis, and flares of fever of unknown origin. Since infancy, she developed occasional head and neck lymphadenopathy; her enlarged adenoids and tonsils were removed at 3 years of age, showing marked polyclonal lymphocytic proliferation. She also experienced non-infectious diarrhea but thrived well. Since 6 years of age, she had had recurrent labial HSV.

At 2.5 years of age, P8 was found to have unmeasurably low levels of serum IgG and IgA, elevated IgM, and low levels of specific antibodies to tetanus, pneumococcal protein vaccine, and MMR, forming the most profound dysregulation of humoral immunity within this cohort. Additionally, P8 was the only patient with low naïve B cells; otherwise, her remaining cellular immunophenotype was unremarkable in the context of the disease.

As a preliminary diagnosis of hyper-IgM syndrome was established, she was started on IgRT, antibiotic, and antifungal prophylaxis, which prevented further severe RTI, controlled the spells of fever, and substantially reduced the lymphadenopathy, but the diarrhea was only partially ameliorated. Labial herpes was well-controlled with acyclovir prophylaxis. At 10 years of age, she developed bronchial asthma, which is well-controlled by inhaled corticosteroids and beta agonists.

The patient currently attends school for children with special needs, after being diagnosed with childhood autism spectrum disorder at 6 years of age.

## Discussion

Our cohort of APDS patients demonstrates a marked clinical heterogeneity, disease severity, and treatment response, corresponding well with previously published cases (8, 9).

The unifying pattern, shared across the cohort, is a combination of frequent upper and lower RTI associated with clinical signs of benign, otherwise unexplained lymphoproliferation, thus, resembling other predominantly antibody deficiencies with combined humoral and cellular defects, such as common variable immune deficiency (CVID) (13). However, uncharacteristically for CVID, the increased infectious susceptibility and lymphoproliferation was pronounced in all patients prior to the fifth year of age, reminiscent of other recently defined monogenic combined immunodeficiencies (CID) with childhood onset, dysgammaglobulinemia, RTI, and autoimmunity, such as cytotoxic T-lymphocyte-associated protein 4 (CTLA4) or lipopolysaccharide-responsive and beige-like anchor protein (LRBA deficiency) (14, 15). Along with recurrent RTI, the majority of patients had chronic lymphadenopathy, persistently enlarged tonsils, adenoids, or splenomegaly since preschool age, resembling autoimmune lymphoproliferative syndrome (ALPS), particularly when associated with autoimmunity. Other hallmark comorbidities were pulmonary damage and gastrointestinal involvement, reminiscent yet again of CVID; however, presenting already in the first decade of life. Interestingly, in four patients, the elevated IgM had misguided the clinical diagnosis toward hyper-IgM syndrome prior to genetic testing.

Over half of the cohort displayed signs of impaired EBV response, consistent with previously reported data (8, 9). Nevertheless, the infection had rather mild features, unlike the often catastrophic EBV-driven lymphoproliferative consequences in other inborn errors of immunity, such as in X-linked lymphoproliferative syndrome (XLP) or interleukin-2-inducible T-cell kinase (ITK) deficiency (16).

Disturbances in serum immunoglobulin values, especially increased IgM, normal or decreased IgG with low IgG2, or disturbed humoral post-vaccination response warrant lymphocyte immunophenotyping in search for the typical CD4 and CD8 naïve T-cell lymphopenia, increased transitional B cells and activation of CD8 T cells associated with APDS. While not universal and sometimes only discrete, such findings may help differentiate APDS from CVID and other CID. Importantly, while all our APDS patients had disturbed B-cell or T-cell compartments, over a third displayed normal or near-normal levels of basic immunoglobulins (both in childhood and adulthood). Therefore, normal immunoglobulin values should not discourage the suspicion of APDS. Conversely, a pure dysgammaglobulinemia with normal cellular immunophenotype is less likely to be seen in APDS.

Curiously, an elevation of serum IgD was observed in three of our patients (in one patient before IgRT, in two patients on IgRT), which has, to our best knowledge, not been described in the literature so far. Present in two related and one unrelated patient, this elevation was persistent, rather than driven by short-term changes in the clinical status of the patients and resolved shortly after initiation of treatment with the specific PI3K inhibitor leniolisib. Increased PI3K signaling has been demonstrated in a model of mevalonate kinase deficiency (17), a disease hallmarked by the elevation of serum IgD. Additionally, the arrest of B-cell development at the stage of naïve and transitional B cells and their relative expansion, seen in APDS, may be partly to blame, as both of these populations express high levels of surface IgD.

Interestingly, among our eight patients with APDS, two suffered from neurodevelopmental impairment manifesting as an autism spectrum disorder and speech delay. The speech delay may be at least in part attributed to the conductive hearing loss due to Eustachian tube dysfunction caused by enlarged adenoids and tonsils. On the other hand, the autism spectrum disorder may be reflective of PI3Kδ role in the central nervous system, as demonstrated in murine models (18).

Historical labeling of APDS patients as a CVID-like or hyper-IgM-like disorder leads to empirical treatment with antibiotics and IgRT, which worked well in tandem with lower infectious morbidity and prevent post-infectious and lymphoproliferation-associated sequelae in all our patients. The mechanism behind suppression of lymphoproliferation with IgRT is not entirely clear; the lower infectious burden and immunomodulatory activity of immunoglobulins may play a role. The understanding of molecular defect underlying the overactivation of the PI3Kδ pathway resulted in tailored treatment with signaling inhibitors such as rapamycin (4), which was at least partially effective in two of our patients. Hematopoietic stem cell transplantation remains the only curative treatment at present time but shows mixed results of >80% 30-year survival but only <40% patients being free of complications (19). Treatment with specific PI3K inhibitors promises best risk–benefit ratio (20) and was beneficial in all our patients. However, due to the small number of cases and short follow-up period, our data are insufficient to draw conclusions about its overall effectiveness and safety.

Our patients received vaccination with inactivated vaccines, including tetanus toxoid, conjugated, or polysaccharide pneumococcal vaccine, inactivated influenza, and live attenuated vaccines (BCG, MMR) without any complications. While the majority mounted an adequate anti-tetanus response, the anti-pneumococcal response was generally low. At least one patient mounted a normal humoral response after MMR; however, the specific IgG levels may wean over time, as they did in other subjects. Therefore, we suggest that APDS patients receive individually tailored vaccination schedules based on the monitoring of specific antibody titers to guide the timing of booster doses. Generally, the timely administration of vaccines prior to the start of immunosuppressive medication would also benefit the antimicrobial competency of the patients.

Of note, half of the cohort, including two patients receiving leniolisib, suffered SARS-CoV-2 infection, although with no or only mild symptoms. Majority of them were able to mount an adequate serologic response. This suggests an undisturbed ability of APDS patients to fend off SARS-CoV-2, despite the impaired specific antibody response noted in some previous works.

Last, APDS classically segregates with autosomal dominant inheritance trait (2). The risk of passing the mutated gene to each child is 50%, and prenatal diagnosis is available for carriers of known mutations. The puzzling occurrence of two affected offsprings (P1 and P2) of healthy parents who do not carry the mutation may be explained by the phenomenon of parental gonosomal mosaicism, recently shown to underlie a number of inborn errors of immunity (21). This observation has important implications regarding the genetic counseling for APDS families.

In summary, we present the early course of APDS in eight patients, which demonstrates that early warning signs of the disease are present since infancy or early childhood. They may be supported by family history suggestive of autosomal dominant inheritance trait and consist typically of frequent respiratory tract infections complicated by early development of structural lung damage, persistent enlargement of lymphatic tissue, and abdominal discomfort or diarrhea. The hallmark laboratory findings are dysgammaglobulinemia, particularly elevated IgM, and normal to low IgG, together with high transitional B cells, and low naïve CD4 and CD8 T cells. Although the differentiation between APDS, CVID, or other CID is challenging, the early diagnosis is critical, especially in the light of the availability of outcome-improving targeted treatment.

## Data Availability Statement

The original contributions presented in the study are included in the article/Supplementary Material, further inquiries can be directed to the corresponding author/s.

## Ethics Statement

Ethical review and approval was not required for the study on human participants in accordance with the local legislation and institutional requirements. Written informed consent to participate in this study was provided by the participants' legal guardian/next of kin. Written informed consent was obtained from the individual(s), and minor(s)' legal guardian/next of kin, for the publication of any potentially identifiable images or data included in this article.

## Author Contributions

MB, AK, and AS conceived the study. MB and AK collected and analyzed the data and co-wrote the manuscript. MB, RZ, and TM provided primary patient data. VK analyzed and interpreted patients' immunophenotype. AS supervised the manuscript preparation. All authors contributed in a substantive manner and reviewed the manuscript.

## Conflict of Interest

The authors declare that the research was conducted in the absence of any commercial or financial relationships that could be construed as a potential conflict of interest.
