# Supplementary material for: Natural Course of Activated Phosphoinositide 3-Kinase Delta Syndrome in Childhood and Adolescence
Source: Front Pediatr. 2021 Jul 19;9:697706. doi: 10.3389/fped.2021.697706 (PMC8326455; doi:10.3389/fped.2021.697706)
Supplement: Supplementary file 1 [file Table_1.DOCX]

|  | **P1** | **P2** | **P3** | **P4** | **P5** | **P6** | **P7** | **P8** |
| --- | --- | --- | --- | --- | --- | --- | --- | --- |
| **Humoral immunity before IgRT** | | | | | | | | |
| **Initial IgG^a^** | Low | Low | ~ 10.6 (6.37-11.05) | ~ 8.18 (7.31-12.75) | Low | ↓ 6.25 (6.37-11.05) | ~ 11.5 (7.65-13.6) | ↓ <0.33 (5.53-10.2) |
| **Initial IgG1^a^** | NA | NA | ~ 9.58 (3.7-10) | ~ 7.80  (3.7-12.8) | NA | ~ 4.61 (3.70-10.00) | ~ 8.81 (4.9-11.4) | NA |
| **Initial IgG2^a^** | NA | NA | ~ 1.41 (0.72-3.4) | ↓ 0.54 (0.98-4.80) | NA | ↓ <0.26 (0.72-3.40) | ↓ 1.45 (1.5-6.4) | NA |
| **Initial IgG3^a^** | NA | NA | ↓ 0.113 (0.13-1.33) | ~ 0.413  (0.18-1.63) | NA | ~ 0.258 (0.13-1.33) | ↑ 1.37 (0.2-1.1) | NA |
| **Initial IgG4^a^** | NA | NA | ↓ <0.007  (0.01-0.84) | ↓ <0.080  (0.04-2.30) | NA | ~ 0.124 (0.01-1.58) | ↓ <0.08 (0.08-1.4) | NA |
| **Initial IgM^a^** | High | High | ~ 1.62 (0.47-1.67) | ↑ 2.28 (0.47-1.18) | High | ~ 1.37 (0.47-1.67) | ~ 1.89 (0.47-1.95) | ↑ 7.04 (0.47-1.67) |
| **Initial IgA^a^** | Normal | Low | ~ 0.97 (0.58-1.16) | ↓ 0.72 (0.91-1.7) | Normal | ~ 0.96 (0.58-1.16) | ~ 2.33 (0.91-2.9) | ↓ <0.07 (0.33-0.91) |
| **Initial IgD** | NA | NA | ↑ 124 (0.0-100.0) | ~ 37.10 (0.0-100.0) | NA | ~ <5.65 (0.0-100.0) | NA | ~ 20.50 (0.0-100.0) |
| **Anti-tetanus^b^** | NA | NA | ~ 0.56 (>0.1) | ~ 3.17 (>0.1) | NA | ~ 0.1 (>0.1) | ~ 0.92 (>0.1) | ↓ 0.05 (>0.1) |
| **Anti-pneumococcus^c^** | NA | NA | ↓ 3.56 (>6) | ↓ 2.4 (>6) | NA | NA | ↓ 5.49 (>6) | ↓ 1.65 (>6) |
| **Anti-MMR^b^ measels  rubella  mumps** | NA | NA | ~ 685.25 (>275.0) ~ 31.5 (>11.0) ~ 67.4 (22.0) | NA | NA | ↓ <50 (>200) ↓ 1.66 (>11) ↓ 5.91 (>22) | ↓ 72 (>200) ↓ 9.62 (>11) ~ 68.75 (>22) | ↓ 21.33 (>275) ↓ <1 (>11) ↓ 0.13 (>22) |
| **Auto-antibodies** | ANA- pANCA+ | ANA- ANCA- | ANA- ANCA- | ANA- pANCA borderline+ ENA- dsDNA- | ANA- ANCA- | ANA- ANCA- ENA- dsDNA- tTG  EM- | ANA- ANCA- ENA- tTG  EM- | ANA- ANCA- |
| **B-cell subpopulations before specific treatment** | | | | | | | | |
| **CD19 abs^d^** | ↓ 0.061 (0.099-0.527) | ↓ 0.08 (0.099-0.527) | ↓ 0.229 (0.27-0.86) | ↓ 0.079 (0.11-0.57) | ~ 0.164 (0.099-0.527) | ↓ 0.136 (0.27-0.86) | ~ 0.13 (0.099-0.527) | ↓ 0.035 (0.27-0.86) |
| **Naïve^e^** | ~ 50 (48.4-79.7) | ~ 71 (48.4-79.7) | ↑ 88 (47.3-77) | ↑ 83 (51.3-82.5) | ~ 76 (48.4-79.7) | ↑ 88 (47.3-77) | ~ 73 (48.4-79.7) | ↓ 45 (51.3-82.5) |
| **Transitional^e^** | ↑ 41 (0.9-5.7) | ↑ 16 (0.9-5.7) | ↑ 71 (4.6-8.3) | ↑ 57 (1.4-13.0) | ↑ 51 (0.9-5.7) | ↑ 61 (4.6-8.3) | ↑ 32 (0.9-5.7) | ↑ 62 (1.4-13) |
| **Class-switched^e^** | ↓ 0.2 (8.3-27.8) | ~ 15 (8.3-27.8) | ↓ 2.5 (10.9-30.4) | ~ 3.3 (8.7-25.6) | ↓ 7.9 (8.3-27.8) | ↓ 2 (10.9-30.4) | ↓ 6 (8.3-27.8) | ↓ 7.4 (8.7-25.6) |
| **MZ-like^e^** | ↓ 5.2 (7.0-23.8) | ~ 8.5 (7.0-23.8) | ↓ 2.7 (5.2-20.4) | ~ 6.8 (4.6-18.2) | ~ 10 (7.0-23.8) | ↓ 4.6 (5.2-20.4) | ~ 7.5 (7-23.8) | ~ 5.3 (4.6-18.2) |
| **Plasmablast^e^** | ↑ 26 (0.4-2.4) | ↑ 3.7 (0.4-2.4) | ~ 1.8 (0.6-5.3) | ~ 1.3 (0.6-6.5) | ↑ 8.9 (0.4-2.4) | ~ 1 (0.6-5.3) | ↑ 5.2 (0.4-2.4) | ~ 1.2 (0.6-6.5) |
| **T-cell subpopulations before specific treatment** | | | | | | | | |
| **CD3 abs^d^** | ↓ 0.568 (0.67-2.38) | ~ 1.009 (0.67-2.38) | ~ 1.498 (1.2-2.6) | ↓ 0.912 (1-2.2) | ↓ 0.48 (0.67-2.40) | ~ 1.287 (1.2-2.6) | ~ 1.058 (0.677-2.383) | ~ 0.689 (1.2-2.6) |
| **CD4 abs^d^** | ↓ 0.258 (0.424-1.509) | ~ 0.629 (0.424-1.509) | ↓ 0.562 (0.65-1.5) | ~ 0.567 (0.53-1.3) | ↓ 0.343 (0.424-1.509) | ↓ 0.326 (0.65-1.5) | ~ 0.543 (0.424-1.509) | ↓ 0.243 (0.62-1.5) |
| **CD8 abs^d^** | ~ 0.244 (0.169-0.955) | ~ 0.354 (0.169-0.955) | ~ 0.832 (0.37-1.1) | ↓ 0.286 (0.33-0.92) | ↓ 0.125 (0.169-0.955) | ~ 0.93 (0.37-1.1) | ~ 0.458 (0.169-0.955) | ↓ 0.359 (0.37-1.1) |
| **TCRγδ^f^** | 4.7 | NA | NA | 3.7 | 2.6 | 4.1 | NA | 9.6 |
| **Treg^g^** | ~ 6.8 (3-9) | ~ 4.8 (3-9) | ~ 4.3 (3-9) | ~ 3.8 (3-9) | ~ 5.1 (3-9) | ~ 3.99 (3-10) | NA | ~ 7.5 (3-9) |
| **Th17 stimulated^g^** | ~ 1.26 (0.47-1.59) | ↓ 0.373 (0.47-1.59) | ~ 0.97 (0.2-1.8) | ~ 0.52 (0.47-1.59) | NA | 0.3 (0.2-1.8) | NA | NA |
| **RTE^g^** | ↓ 3.4 (7-100) | ~ 17 (7-100) | ↓ 29 (41-81) | ↓ 17 (31-81) | ~ 13 (7-100) | ↓ 4.6 (41-81) | ~ 8.7 (7-100) | ↓ 19 (41-81) |
| **Naïve CD4^g^** | ↓ 7.4 (16-100) | ~ 31 (16-100) | ~ 39 (46-99) | ↓ 35 (37-97) | ~ 22 (16-100) | ↓ 7 (46-99) | ↓ 12 (16-100) | ↓ 23 (46-99) |
| **HLA-DR+ CD4^g^** | ↑ 38 (0-20) | ~ 8.3 (0-20) | ~ 7.2 (0-20) | ~ 12 (0-20) | ~ 10 (0-20) | ↑ 33 (0-20) | ~ 15 (0-20) | ~ 14 (0-20) |
| **Naïve CD8^h^** | ~ 21.5 (6-100) | ~ 30 (6-100) | ~ 40 (16-100) | ↓ 19 (20-95) | ~ 9.5 (6-100) | ↓ 2.4 (16-100) | ~ 23 (6-100) | ↓ 3.6 (16-100) |
| **HLA-DR+ CD8^h^** | ↑ 31 (0-20) | ↑ 31 (0-20) | ~ 13 (0-20) | ↑ 34 (0-20) | ↑ 29 (0-20) | ↑ 52 (0-20) | ↑ 36 (0-20) | ↑ 33.1 (0-20) |
